# Mutational load of the mitochondrial genome predicts pathological features and biochemical recurrence in prostate cancer

**DOI:** 10.18632/aging.101044

**Published:** 2016-10-05

**Authors:** Anton M.F. Kalsbeek, Eva F.K. Chan, Judith Grogan, Desiree C. Petersen, Weerachai Jaratlerdsiri, Ruta Gupta, Ruth J. Lyons, Anne Maree Haynes, Lisa G. Horvath, James G. Kench, Phillip D. Stricker, Vanessa M. Hayes

**Affiliations:** ^1^ Laboratory for Human Comparative and Prostate Cancer Genomics, Genomics and Epigenetics Division, Garvan Institute of Medical Research, Darlinghurst, NSW 2010, Australia; ^2^ School of Medical Sciences, University of New South Wales, Randwick, NSW 2031, Australia; ^3^ Department of Tissue Pathology and Diagnostic Oncology, Royal Prince Alfred Hospital, Camperdown, NSW 2050, Australia; ^4^ Central Clinical School, Sydney Medical School, University of Sydney, Camperdown, NSW 2050, Australia; ^5^ Cancer Research Division, The Kinghorn Cancer Centre/Garvan Institute of Medical Research, Darlinghurst, NSW 2010, Australia; ^6^ Chris O'Brien Lifehouse, Missenden Road, Camperdown, NSW 2050, Australia; ^7^ Department of Urology, St. Vincent's Hospital, Darlinghurst, NSW 2010, Australia

**Keywords:** prostate cancer, mitochondrial genome, prognostic biomarkers, mutational load

## Abstract

Prostate cancer management is complicated by extreme disease heterogeneity, which is further limited by availability of prognostic biomarkers. Recognition of prostate cancer as a genetic disease has prompted a focus on the nuclear genome for biomarker discovery, with little attention given to the mitochondrial genome. While it is evident that mitochondrial DNA (mtDNA) mutations are acquired during prostate tumorigenesis, no study has evaluated the prognostic value of mtDNA variation. Here we used next-generation sequencing to interrogate the mitochondrial genomes from prostate tissue biopsies and matched blood of 115 men having undergone a radical prostatectomy for which there was a mean of 107 months clinical follow-up. We identified 74 unique prostate cancer specific somatic mtDNA variants in 50 patients, providing significant expansion to the growing catalog of prostate cancer mtDNA mutations. While no single variant or variant cluster showed recurrence across multiple patients, we observe a significant positive correlation between the total burden of acquired mtDNA variation and elevated Gleason Score at diagnosis and biochemical relapse. We add to accumulating evidence that total acquired genomic burden, rather than specific mtDNA mutations, has diagnostic value. This is the first study to demonstrate the prognostic potential of mtDNA mutational burden in prostate cancer.

## INTRODUCTION

Prostate cancer is the most commonly diagnosed malignancy in men world-wide and a major cause of cancer death [1,2]. As the global population is aging, so is the total health burden of prostate cancer increasing. Clinical management is constraint by significant heterogeneity in disease course from indolence to mortality [3]. Currently, elevated serum prostate-specific antigen (PSA) and/or abnormal digital rectal examination (DRE) are used as the rationale for a needle biopsy and histopathological diagnosis and Gleason scoring, which is a grading system based on prostate gland histological architecture and used for prognosis [4,5]. Although the only biomarker in routine clinical use, PSA testing has led to over-diagnosis and overtreatment with no definitive mortality benefit [6,7]. Ultimately, the effective treatment of prostate cancer requires novel molecular biomarkers that provide early detection of lethal disease associated with metastasis, while appropriate detection of indolent disease is required to prevent overtreatment and, in turn, improve patient quality of life [8].

Genomic changes are significant contributors to prostate cancer from inherited risk [9] to somatic events initiating and driving tumor progression [10]. Although no definitive disease driver mutations have been identified, exome and whole genome sequencing efforts have identified several prostate cancer subgroups based on genome profiling [11–13]. However, these studies have focused on the nuclear genome, largely ignoring the maternally inherited mitochondrial genome and its potential as a prostate cancer biomarker.

The significance of the mitochondrial genome can be directly attributed to mitochondrial function, in particular the generation of the majority of cellular energy in the form of adenosine triphosphate (ATP) through oxidative phosphorylation (OXPHOS) of glucose, as well as significant roles in the regulation of apoptosis and calcium homeostasis. Cells have multiple mitochondria, each containing several copies of the mitochondrial genome. The 16,569 base circular haploid genome encodes for 37 genes; 13 protein coding, 2 ribosomal rRNA and 22 transfer tRNAs. A 10-fold higher mutation rate than nuclear DNA, it is not surprising that mutations in the mtDNA have been associated with several cancers, including prostate cancer [14–17]. These sporadic variants may become fixed or lost over time, creating a state of heteroplasmy. The assumption is that a variant need to reach a frequency threshold to have an impact on cell function [18,19].

Including 50 variants in the mtDNA variation database MITOMAP, as at 9^th^ of April 2016, a total of 380 prostate cancer associated somatic mtDNA mutations have been reported [16,20–23]. Most recently, McCrow et al., 2016 highlighted that prostate cancer presentation is more likely characterized by the total accumulation of mtDNA mutations rather than specific mtDNA mutations [22], while Ju et al., 2014, looking at mtDNA mutations across a range of cancer tissue types, suggested that functionally deleterious mtDNA mutations were more likely to be heteroplasmic as a result of negative selection [20]. These studies provide evidence for the pathogenic impact and diagnostic value of somatic mtDNA mutations, emphasize the need for further deep whole mitochondrial prostate cancer genome sequencing, and importantly highlight the need for associated clinical follow-up data to assess for prognostic value.

In this study, we compared normal-prostate tumor paired mitochondrial genomes from 115 men having undergone a mean follow-up of 107 months post radical prostatectomy. As well as assessing the biological impact of somatic variants on mitochondrial function and tumor formation, we aimed to correlate the mtDNA mutational burden with clinical impact including diagnosis, defined as pathological presentation, and patient outcomes, defined as biochemical and clinical relapse.

## RESULTS

### Prostate cancer patient clinical and pathological characteristics

All 115 patients presented with prostate cancer and elected for surgical removal of the prostate gland, with a mean of 107 months (range 24-150 months) follow-up. Patient and pathological characteristics may be found in Table 1. In brief, the mean age at radical prostatectomy was 61 years (range 46 to 75 years), with pre-operative PSA levels ranging from 2.43 to18.6 ng/ml. Since our cohort is biased towards more aggressive disease presentation, defined by a Gleason score ≥7 (89/115, 77%), a substantial number of patients (41/115, 36%) had relapsed within three years (range 1 to 126 months) post-surgery. Radical prostatectomy was non-curative in eight patients presenting with advanced stage prostate cancer, two with reported mortality, while eight patients have advanced to metastatic disease. Pathological reassessment of the core biopsy from which DNA was extracted for this study corresponded with the total and primary Gleason score at surgical diagnosis in 46% (53/115) and 71% (82/115) of cases respectively, further detailed in Supplementary Table 1, while tumor purity estimations averaged 57% (range 5% to 85%).

**Table 1:** Patient and prostate tissue core characteristics

	Gleason 6	Gleason 7	Gleason 8-10	**ALL**
*Patient characteristics*				
*Prostate tissue core characteristics*				
**Radical Prostatectomy (RP)**				
Total patient numbers	26	40	49	115
Mean age in years (range)	46 (58-67)	63 (49-74)	61 (50-75)	61 (46-75)
Mean PSA in ng/mL (range)	6.35 (2.43-12)	8.36 (2.5-16.4)	8.61 (3.5-18.6)	8 (2.43-18.6)
**Outcomes**				
Total follow up in months (range)	105 (51-142)	122 (63-146)	83 (24-150)	107 (24-150)
Nil relapse^1^	24	27	23	74
Relapse totals	2	13	26	41
Non curative RP	0	0	8	8
BCR only	2	11	10	23
BCR & metastasis^2^	0	2	6	8
Death from PCa	0	0	2	2
Months to recorded relapse (range)	59 (36-82)	54 (8-126)	1 (21-78)	33 (1-126)
***Prostate tissue core characteristics***				
**Pathology**				
Total/Major Gleason score matching RP	18/26	22/28	13/28	53/82
Mean est. % tumor purity (range)	47 (5-75)	60 (20-80)	60 (25-85)	57 (5-85)
**mtDNA mutational spectrum**				
Total no SNVs identified	6	27	43	76
Patients with SNVs^3^	4	19	27	50
Mean SNV count per patient (range)	0.23 (0-2)	0.68 (0-3)	0.88 (0-4)	0.66 (1-4)
Total non-synonymous SNV identified	3	10	13	26
Mean absolute SNV allele frequency	0.47 (0.17-0.96)	0.44 (0.15-0.89)	0.49 (0.12-1)	0.46 (0.12-1)
Mean adjusted SNV allele frequency^4^	0.65 (0.28-1.37)	0.77 (0.24-1.79)	0.83 (0.17-1.43)	0.75 (0.17-1.79)
Mean cumulative SNV allele frequency	1.35 (0.29-3.25)	1.24 (0.28-2.81)	1.94 (0.29-3.25)	1.42 (0.28-3.25)

Abbreviations: RP, radical prostatectomy; BCR, biochemical recurrence; PCa, prostate cancer; SNVs, single nucleotide variants

1 Two recorded deaths not defined as a result of prostate cancer

2 Bone or visceral metastasis

3 One or more mtDNA SNVs observed

4 Adjusted for pathologically estimated tumor purity

### Mitochondrial haplogroup distribution and prostate cancer risk association

Previous studies have suggested that inherited mitochondrial genome variation which defines population-specific haplogroups, may be associated with prostate cancer risk. Specifically, a study of 221 white North American men showed increased risk of haplogroup U for developing PCa [odds ratio 1.95] [24]. Sequencing complete mitochondrial genomes from blood, we found the haplogroup frequencies did not deviate significantly (*p* = 0.95 by two-sided Fischer's exact test) from that expected for an Australian European-ancestral population [25] with haplogroup H most commonly represented at 43% and 44% for expected and observed frequencies respectively, followed by U (both 14%), T (both 9%), J (11% versus 9%) and K (8% versus 7%). Haplogroups were also not associated with specimen Gleason score (*p* = 0.81 by two-sided Fischer's exact test). Patient specific haplogroups are depicted in Supplementary Table 1. Our study is in agreement with a more recent report that suggests that common European-derived mtDNA haplogroups are not correlated with increased prostate cancer risk [26,27] while the potential association between the earliest diverging L0 African-derived mtDNA haplogroups and aggressive prostate cancer [22] remains to be confirmed.

### Spectrum and frequency of acquired prostate cancer specific mitochondrial genome variation

**Figure 1:**
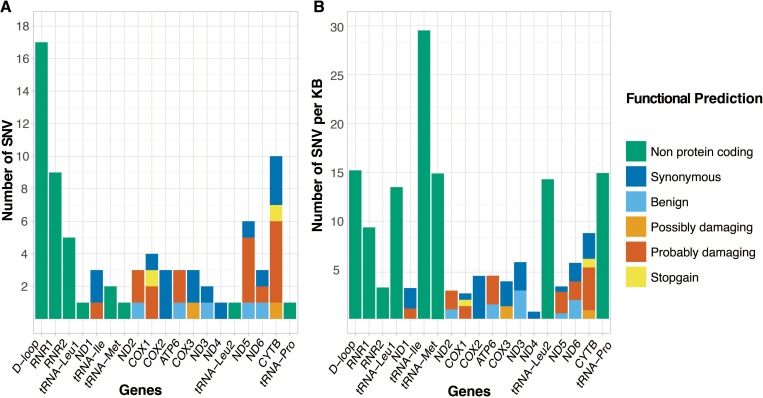
**Number of somatic single nucleotide variants (SNVs) distributed over the gene, control and non-protein coding regions of the mitochondrial genome,** defined as (A) absolute number of SNVs and (B) number of SNVs scaled by region length (SNVs per 1000 bases). Genes/regions are represented in order of appearance on the mitochondrial genome, with genes colored by functional prediction of SNV.

Sequencing mtDNA from matched tumors, we identified a total of 76 somatic single nucleotide variants (SNVs) in 50 patients (43.5%) (Table 1). A single variant recurred in three patients at position mt.16093, resulting in 74 unique variants (see Supplementary Table 2 for full list of variants). While sequencing was performed using the Ion Torrent PGM instrument, we used the Illumina HiSeq X Ten platform to validated 11/12 (91.7%) unique SNVs within 16 randomly selected tumor-normal sample pairs. The single SNV not identified in the HiSeq data represented a loss of a heteroplasmic low allele frequency (0.17) SNV and is most likely to be a discrepancy in variant calling between the platforms. We retained the SNV for our analysis. Compared with publicly available data, 91.9% (68/74) of the observed unique variants are novel to prostate cancer, 86.5% (64/74) novel to any cancer, and 77.0% (57/74) novel to any disease. Although distributed across the entire mitochondrial genome, we found a predominance of variation within the control region and ribosomal RNA genes (Figure 1A). When correcting for length, we found a greater, though not significant, frequency of variation within the non-protein coding than protein coding regions (*p* = 0.013 by two-sided Fischer's exact test) (Figure 1B). This proved to be significant for the D-loop and the tRNA regions (*p* = 0.0044 by two-sided Fischer's exact test), as previously reported [16]. Of the 41 SNVs mapping to protein coding regions, 26 directly alter an amino acid (non-synonymous mutation) of which 21 have biological potential defined by termination of translation (n=2) or by PolyPhen-2 computational prediction as either possibly (n=2) or probably damaging (n=17). The gene coding for protein Cytochrome b was affected most by damaging variants, both in absolute numbers and when corrected for gene length. Somatic SNVs further showed a predominance of pyrimidine nucleotide transition events, 54% C>T and 24% T>C in line with what was reported previously [20]. To determine the potential of SNVs to impact mitochondrial function (>70% variant frequency), we corrected for tumor purity estimates, resulting variant frequencies ranged 0.17-1.79, (average 0.75). Following this adjusted variant frequency the cumulative variant frequency (CVF) in each patient with variants ranged 0.28-3.25 (average 1.42). Investigating a previously reported ~3.4 kb deletion variant, long range PCR did not show this deletion to be detectable in any of our patients [28].

### Correlating somatic mtDNA variation with clinical presentation and outcomes

**Figure 2:**
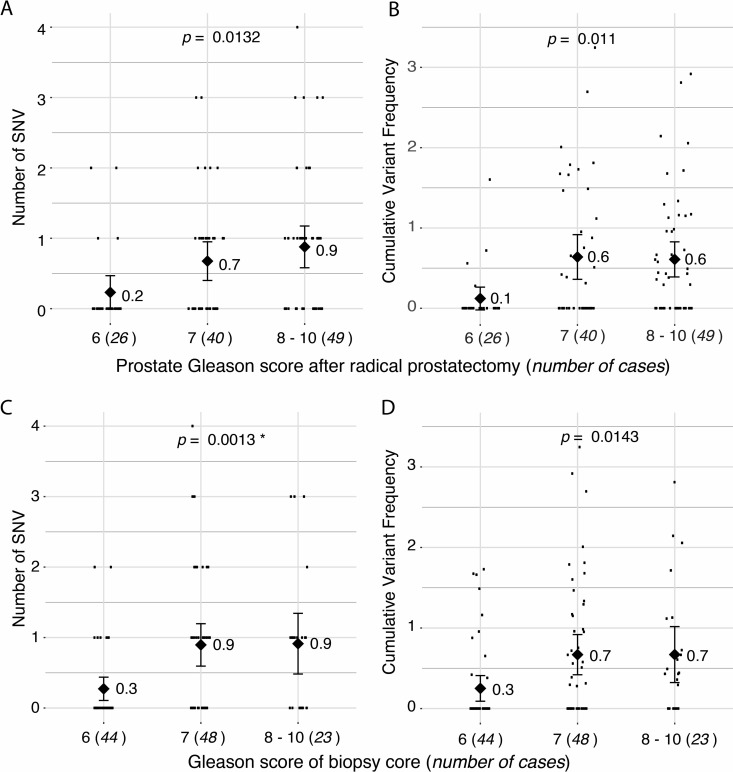
Correlation of number of somatic single nucleotide variants (SNVs) with Gleason score (**A**) Total number of somatic SNVs per tumor and (**B**) cumulative variant frequency (CVF) categorized by Gleason score at radical prostatectomy. (**C**) Total number of somatic SNVs per tumor and (**D)** CVF categorized by Gleason score of screened biopsy core. *P*-values represents linear model of SNV predicting pathology score. Diamonds and numbers represent the mean of each group.

While we found no single somatic mtDNA mutations common between patients, we observed a diagnostic and prognostic correlation with the total somatic variant burden and for the CVF per patient. Specifically, an increase in total number of somatic mtDNA SNVs and CVF was significantly associated with high-risk disease defined as an increased Gleason score at radical prostatectomy *p* = 0.0132 (Figure 2A) and *p =* 0.011 (Figure 2B), respectively, and within the sampled biopsy core *p* = 0.0013, (Figure 2C) and *p =* 0.0143 (Figure 2D), respectively, with *p*-values estimated from ANOVA models. Analyses for subgroups of variants showed a significant predictive value for number of non-synonymous variants (*p* = 0.034 by ANOVA) and synonymous and non-coding variants (*p* = 0.018 by ANOVA). We point out that, when adjusting for multiple testing using the conservative Bonferroni correction, all six tests drop below the significance threshold at the familywise alpha level of 5% (α = 0.05/7 = 0.0071). However, we note these tests are not independent, with two pairs of highly correlated variables, namely: number of SNV and CVF, and Gleason Scores at prostatectomy and of the biopsy cores.

**Figure 3:**
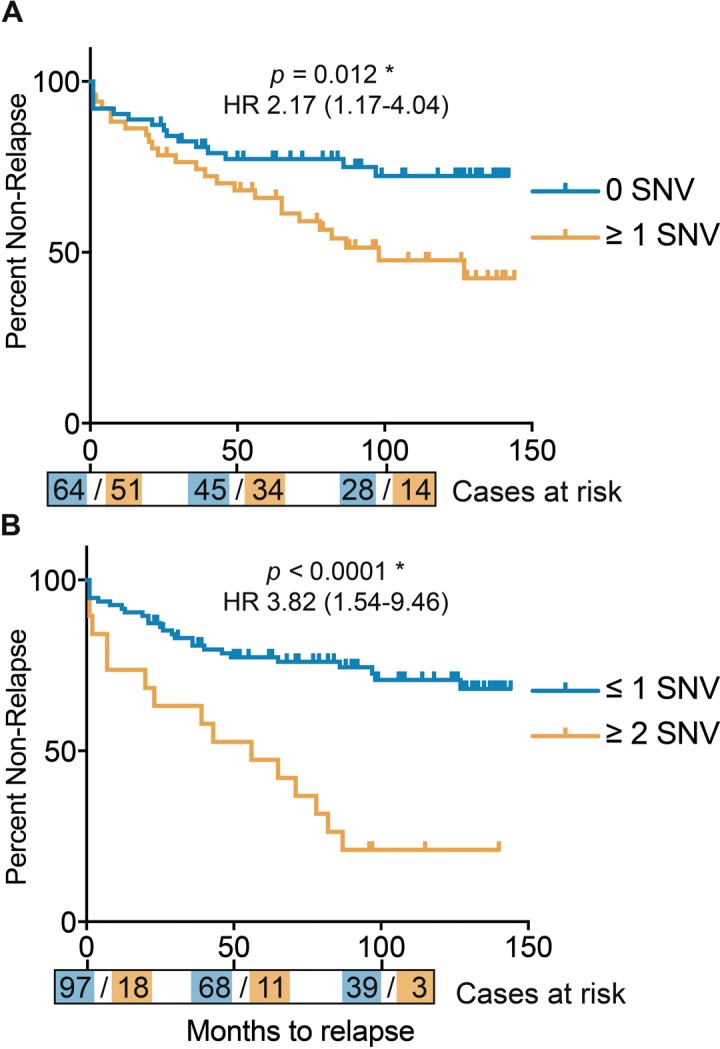
Kaplan Meier curves for disease relapse after surgery, defined by total number of mtDNA single nucleo-tide variants (SNVs) (**A**) Patients divided by zero or any mtDNA SNVs. (**B**) Patients divided by 2 or more and less than 2 mtDNA SNVs. Time in months, with number of cases per mtNDA SNV category represented at time-points 0, 50 and 100 months.

**Figure 4:**
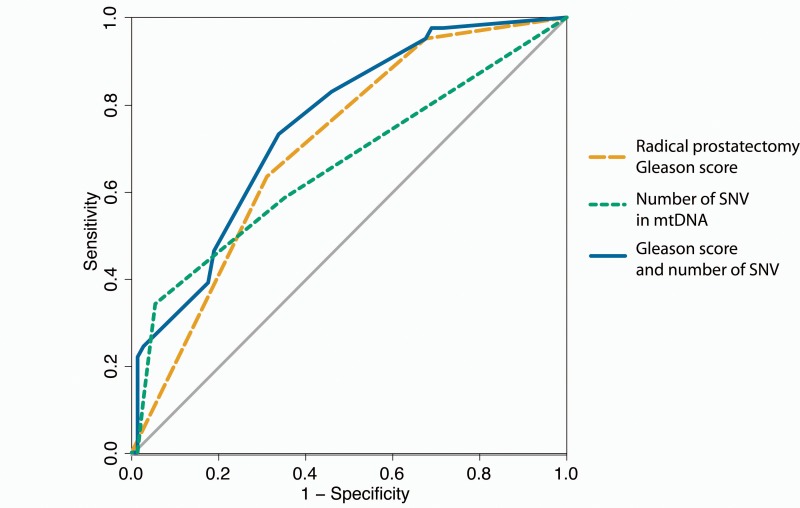
ROC curves for predicting disease relapse In orange disease relapse predicted by Gleason score of the radical prostatectomy alone (AUC = 0.704), green for number of single nucleotide variants (SNVs) in mtDNA alone (AUC = 0.659) and in blue the Gleason score combined with the number of mtDNA SNVs (AUC = 0.752).

When investigating the correlation between Gleason score and tumor purity we found a strongly significant correlation (*p* = 1.6e-5 by ANOVA for the linear model of: Gleason Score ~ Purity), consequently, the number of SNVs also correlates significantly with tumor purity (*p* = 0.003 by ANOVA for linear model of: SNV number ~ Purity). Additionally, patients with one or more somatic SNVs were significantly more likely than patients without any somatic mtDNA variants to present with a disease relapse (*p* = 0.012, hazard ratio (HR) 2.17 (confidence interval (ci) 1.17 – 4.04) by log rank test, Figure 3A), with risk for relapse increasing when considering 2 or more SNVs (*p* < 0.0001, HR 3.82 (ci 1.54 – 9.46) by log rank test (Figure 3B). As Gleason score is currently the most significant predictor for prostate cancer, we determined the power of total mtDNA variation burden to predict disease relapse in our study either alone and in combination with Gleason score, suggesting that number of mtDNA SNVs slightly improves relapse prediction in combination with Gleason score (*p* = 0.085 by DeLong test for area under curve (AUC)) (Figure 4).

## DISCUSSION

In this study, we contribute 68 novel prostate cancer associated mtDNA mutations to the growing somatic catalog, while confirming six others. Taken together, roughly 3% of the total mitochondrial genome has now been shown to have mutagenic potential during prostate tumourigenesis. Accounting for previous study biases towards the restricted analysis of the hypervariable regions (HVRs) within the control region (D-loop region) of mtDNA, the greatest overall impact appears to be within the smaller 1.2 kb non-coding portion of the genome, observing 14 SNVs per kb in our study. We confirm a previously reported bias towards mutational events within the tRNAs, reporting 8.7 SNVs per kb. We predict 32% (24/74) of our identified mutations to have biological potential, with roughly half (9/24) present in sufficient frequency (>70%) to alter mitochondrial function. Further biological studies are required to determine the overall impact of these somatic mutations on energy metabolism, specifically their potential to drive tumor respiration from one of oxidative phosphorylation to aerobic glycolysis [29], a common cancer phenomenon known as the Warburg effect [30].

The unique properties of mtDNA including its small size, high copy number, susceptibility to mutations and heteroplasmic populations has spearheaded investiga-tions into the clinical significance of somatic mtDNA variation. A recent study suggested that, it is more likely the accumulation of mitochondrial mutations across the entire genome rather than specific mutations that contribute to prostate cancer progression [22]. The latter study showed an association between the mutational load, which is the total number and frequency of mtDNA somatic mutations, and higher Gleason score at diagnosis. We concur that the number and cumulative frequency of SNVs in the mitochondrial genome appears to be an independent predictor for prostate cancer presentation, with significant diagnostic potential for aggressive disease. While we confirm a diagnostic value to the number and frequency of somatic mtDNA SNVs based on a single core interrogation, we do not provide support for the previously reported 3.4 kb mtDNA deletion variant. It should be noted, however, that Mitomics^TM^ proposes a minimum five-specimen requirement to achieve adequate sensitivity for early prostate cancer detection.

Historically studies focused on the identification of molecular biomarkers for prostate cancer have lacked prognostic potential. This is the first study, to our knowledge, to investigate the prognostic role of somatic mtDNA in prostate cancer, supported by the availability of extensive long-term clinical follow-up. We demonstrate that an increase in the number of mtDNA somatic SNVs, both the absolute and CVF is significantly correlated to disease relapse and that this prediction appears to improve stand alone Gleason score based risk prediction. The clinical significance of identifying patients prone to relapse has recently been highlighted by the reported benefit from salvage therapy at the earliest opportunity [31]. Associating an increase in number of mtDNA somatic SNVs with both aggressive disease at presentation and poor outcomes, questions whether we observe a limitation to the number of mutagenic events a cell can tolerate. Although we observed a single individual with four SNVs, the upper limit in our study appears to be three mutagenic events. Further cataloging of prostate cancer associated mitochondrial mutations is required not only to determine the spectrum and combination of oncogenic variants, as well as the upper limits for cellular viability, but importantly, to provide significant clinical potential.

## METHODS

### Patient selection and sample preparation

Patients were recruited and consented and biospecimens biobanked according to St Vincent's Hospital Human Research Ethics approval #HREC/12/SVH/323 at time of surgery. Study inclusion criteria was as follows; (i) a clinicopathological confirmed diagnosis of prostate cancer, (ii) no pre-operative treatment received (including hormone deprivation therapy, chemo or radio-therapy), (iii) a follow up of more than 24 months post radical prostatectomy, and (iv) the availability of a fresh frozen biopsy core with a matching blood sample. Biopsy cores were processed using cryostat-assisted frozen section sample preparation for pathology review (minimally two pathologists JG, RG, and/or JGK) to ensure tumor presence, evaluate the core pathology for percentage adenocarcinoma cells and Gleason score, and maximize for tumor purity during DNA extraction. DNA was extracted from matching blood and prostate tissue using standard methods (Qiagen) and made available under additional site-specific St Vincent's Hospital Human Research Ethics approval #HREC/15/SVH/227 for whole mitochondrial genome sequencing. Based on these criteria a total of 115 patients were included in this study with clinic-pathological data related to age, PSA level and Gleason score at diagnosis, as well as follow-up with clinical and biochemical relapse data summarized in Table 1 and further elaborated in Supplementary Table 1.

### Mitochondrial genome sequencing

To ensure mitochondrial genome specificity and thereby avoiding amplification of nuclear copies of mitochondrial-derived DNA (NuMTs), we performed a dual-amplicon long-range pre-amplification of the mitochondrial genome prior to sequencing on the Ion Torrent PGM instrument, as previously described [22]. In brief, after quantification, the 7.2 kilobase (kb) and 9.7 kb amplified products were combined in a 7:13 ratio, 200 bp libraries prepared using the Ion Xpress Plus Fragment Kit and Ion Xpress™ Barcode Adaptors (ThermoFisher), and six to eight matched normal-tumor pairs pooled and run on a 318v2 Chip. We generated per patient an average of 80 Megabytes (MB) of data, with an average coverage depth of more than 2000 X per mitochondrial genome.

### Variant calling and annotation

Sequencing data were analyzed using the Ion Torrent suite v5.0.2.1. Specifically, sequencing reads were quality trimmed, aligned to the revised Cambridge Reference Sequence (rCRS, accession number: NC_012920) [32] and variants called using the Torrent Variant Caller v5.0.2.1 with customized parameters optimized for the mitochondrial genome. Somatic variants were identified by patient matched tumor-normal (blood) mitochondrial genome comparison, using an allele frequency difference greater than 10%. Variant annotation was performed using ANNOVAR [33] and functional impact was predicted using PolyPhen-2 [34]. In addition to single nucleotide variants (SNVs) and indels (small insertions and deletions), we used a previously published long-range 3.8 kb targeted amplification method [22] to screen for the 3379 bp mtDNA deletion suggested to be associated with prostate cancer in a north American cohort [28].

### Variant validation

A total of 16 normal-tumor pairs (14% of the study) were randomly selected to undergo whole genome paired-end 2 × 150 bp sequencing using the Illumina HiSeq X Ten platform. A mean coverage of 44X and 71X was generated for the blood and tumor, respectively, resulting in mitochondrial genome coverage of roughly 3000 X and 14000 X, respectively. Mitochondrial sequences were trimmed and filtered and aligned to rCRS using bwa-mem v0.7.12 [35]. SNVs and indels were called using Strelka [36] and manually compared to sample matched SNVs/indels using the Ion Torrent PGM. Although several somatic indels were called using the Ion Torrent data, none were validated and no additional indels observed using the HiSeq X ten data. Conversely, 11/12 Ion Torrent called SNVs were HiSeq X ten validated and therefore only SNVs were used in subsequent mtDNA somatic mutation analyses.

### Clinical correlation and statistical analysis

Clinically relevant data was extracted using CanSto ®, an in-house clinical database manager. Presentation at diagnosis was defined by three Gleason score categories (< 7, equal to 7, and > 7). Disease relapse was defined in this study as; non-curative surgery or a rise in PSA >0.2 ng/mL in two consecutive measurements (biochemical recurrence) either with or without the presence of distal visceral or bone metastasis, and/or associated death. Fisher's exact test was used to test for difference in expected versus observed haplogroups, for difference in presentation at diagnosis and for testing expected versus observed variants in mtDNA regions. ANOVA linear regression models were used to test for clinical correlation, either Gleason score at radical prostatectomy or for the specific biopsy core, with mtDNA somatic mutation rate defined as SNV number or CVF. When more than two statistical tests were performed on subgroups, Bonferroni multiple comparison correction was performed using an alpha of ɑ = 0.05. Statistical analysis and figures were generated in R (R Foundation, Vienna, Austria) using the ggplot2 package [37], with receiver operating characteristic (ROC) curves and area under the curve (AUC) displayed and analyzed using pROC [38]. Prism 7.0 was used to draw and test survival differences using Kaplan Meier Curves.

## SUPPLEMENTARY MATERIAL TABLES






