# Supplementary material for: Mutational load of the mitochondrial genome predicts pathological features and biochemical recurrence in prostate cancer
Source: Aging (Albany NY). 2016 Oct 5;8(11):2702–11. doi: 10.18632/aging.101044 (PMC5191864; doi:10.18632/aging.101044)
Supplement: Supplementary file 1 [file aging-08-2702-s001.pdf]

SUPPLEMENTARY MATERIAL

Please browse the links in Full Text version of this manuscript to see Supplemental Tables.

Supplemental Table 1. Patient epidemiological and clinical information. RP type, PSA at presentation, pathology details, age at RP, follow up time, outcome

Supplemental Table 2. Somatic variants. Allele frequency, specimen details, Annotation, functional prediction
